# False Positive B-Cells Crossmatch after Prior Rituximab Exposure of the Kidney Donor

**DOI:** 10.1155/2016/4534898

**Published:** 2016-04-28

**Authors:** Judith Desoutter, Marie-Joëlle Apithy, Ségolène Bartczak, Nicolas Guillaume

**Affiliations:** Department of Histocompatibility, Amiens University Medical Center, 80000 Amiens, France

## Abstract

Crossmatching is essential prior to kidney transplantation to confirm compatibility between the donor and the recipient, particularly to prevent acute antibody-mediated rejection. An unexpected positive crossmatch may be obtained in recipients with an autoimmune disease or preexisting antibodies not detected by single-antigen bead array due to complement interference or who have been previously treated by desensitization protocols such as rituximab, antithymocyte globulin, or intravenous immunoglobulins. We report donor and recipient investigations that revealed unexpected positive B-cells crossmatch, probably due to donor cells, as the donor had received rituximab therapy shortly before organ harvesting, in a context of severe idiopathic thrombocytopenic purpura. We consequently detected unexpected Class II IgG complement-dependent cytotoxicity for all sera tested. Other laboratory investigations failed to elucidate the reasons for this recipient-related positivity.

## 1. Introduction

The presence of preformed donor-specific antibodies (DSA) directed against human leukocyte antigens (HLA) interferes with kidney transplantation, as it is associated with all types of antibody-mediated rejection. Prior to transplantation, recipients are therefore routinely screened for preformed anti-HLA antibodies and prospective crossmatches are performed by conventional complement-dependent cytotoxicity crossmatch (CDC-XM) techniques, but also by flow-cytometry-based methods [1, 2].

The CDC-XM method is based on incubation of donor isolated B- and T-lymphocytes with recipient serum. The presence of anti-HLA antibodies in serum, targeting donor HLA antigens, induces donor cells complement-dependent cytotoxicity. Positive T-cells IgG-CDC-XM constitutes a contraindication for transplantation. Some centers have extended this contraindication to positive B-cells IgG-CDC-XM.

Positive CDC-XM can be observed in other situations, notably in recipients with an autoimmune disease [3] or preexisting antibodies not detected by single-antigen bead array due to complement interference [4] or previously treated by desensitization protocols such as rituximab (RTX), antithymocyte globulin, and intravenous immunoglobulins [5]. In the prospective setting, an unexpected positive CDC-XM must be rapidly documented to avoid nonaccessibility to the transplant.

We report donor and recipient investigations revealing unexpected positive B-cells crossmatch, probably due to donor cells.

## 2. Case Report

A 46-year-old woman with end-stage kidney disease was considered for first kidney transplantation.

HLA-A^*∗*^30, HLA-B^*∗*^13, HLA-B^*∗*^40, HLA-DRB1^*∗*^04, HLA-DRB1^*∗*^13, HLA-DQB1^*∗*^03, and HLA-DQB1^*∗*^06 genotyping was performed with PCR-SSO genotyping test (One Lambda, Canoga Park, CA). A high-definition LABScreen® single-antigen Class I and Class II assay (One Lambda, Canoga Park, CA) was prospectively performed on the LABScan100® flow cytometer (Luminex Corporation, Austin, TX) to determine the specificity of anti-HLA IgG antibodies. A positive result was defined as mean fluorescence intensity (MFI) greater than 1,000. This assay revealed the presence of anti-A2, anti-A10, anti-A24, anti-A25, anti-A26, anti-A28, anti-A29, anti-A32, anti-A34, anti-A43, anti-A66, anti-A68, anti-A69, anti-A74, anti-B8, anti-B14, anti-B17, anti-B38, anti-B48, anti-B55, anti-B57, anti-B58, anti-B59, anti-B60, anti-B64, anti-B65, anti-B70, anti-B71, anti-B72, anti-B81, anti-B82, anti-Cw7, anti-Cw17, and anti-DR7 antibodies.

A potentially suitable ABO-compatible organ was found with HLA-A^*∗*^03, HLA-A^*∗*^30, HLA-B^*∗*^35, HLA-B^*∗*^49, HLA-C^*∗*^03, HLA-C^*∗*^04, HLA-DRB1^*∗*^04, HLA-DRB1^*∗*^13, HLA-DQB1^*∗*^03, HLA-DQB1^*∗*^03, HLA-DPB1^*∗*^03, and HLA-DPB1^*∗*^15 status. The recipient had no identified donor-specific antibodies (DSA). A prospective CDC-XM was performed with selected nodal T- and B-donor cells (Fluorobeads® T and B, One Lambda) to distinguish anti-HLA Class I and II antibodies, with or without recipient serum pretreated by dithiothreitol (DTT) to distinguish IgG and IgM antibodies. We used as positive controls anti-HLA Class I (# hla-c1, Invivogen, San Diego, USA) and anti-HLA Class II (# hla-c2, Invivogen, San Diego, USA) controls to highlight the quality of the cell suspension, respectively, enriched for T- or B-cells in the corresponding well.

We detected an unexpected Class II IgG complement-dependent cytotoxicity for all sera tested, enhanced by DTT treatment according to the ASHI scoring system (1 and 2 as negative, 4 as 30–49%, 6 as 50–79%, and 8 as 80–100% lysed lymphocytes (see Table 1)) and also in the B-cells negative control well (serum pool from donors which shows no cytotoxic reactions in the lymphocytotoxicity test, Bio-Rad, CA). Because of the unexplained strongly positive Class II IgG, transplantation was not performed by our center.

To test the hypothesis that positive CDC-XM reflects the presence of unidentified antibodies directed against the donor, we performed investigations on the recipient, which failed to provide any explanation for the positive CDC-XM:

- No treatment to prevent acute rejection before transplantation.
- Negative auto-CDC-XM between cells (B- and T-lymphocytes) and recipient serum in accordance with the lack of a documented autoimmune disease.
- Absence of detection of preexisting antibodies due to a complement interference phenomenon by testing sera after EDTA pretreatment, as previously described (0.1 M solution of disodium EDTA, Sigma-Aldrich, St. Louis, MI, at pH = 7.4 diluted 1 : 10 in serum and incubated for 10 min before LABScreen single-antigen testing) [4].

We also performed a donor auto-CDC-XM with donor serum collected on the day of organ harvesting. This assay was positive for B-cells negative control well again, for B-cells with donor serum, and was also enhanced by sera DTT pretreatment. Detailed review of the donor's medical history revealed a diagnosis of severe idiopathic thrombocytopenic purpura, refractory to treatment by corticosteroids, IV immunoglobulins, splenectomy (performed six months before organ harvesting), eltrombopag, and romiplostim. RTX therapy (only one injection) was initiated 12 days before the donor's death.

## 3. Discussion

CDC-XM reveals the functional potential of anti-HLA antibodies to activate complement and can be used to guide the decision to perform transplantation. We report a case of false-positive B-cells CDC-XM due to donor RTX therapy prior to organ harvesting.

In the case of RTX therapy, CDC-XM positivity is restricted to B-cells [6]. False-positive CDC-XM may be detected at low RTX concentrations (0.02 *μ*g/mL) corresponding to blood concentrations observed several months after the last infusion [5]. Moreover, pretreatment of serum by DTT reduced lysis triggered by adding RTX. However, in this case of donor (and not recipient) RTX therapy revealed an opposite effect with complement-dependent cytotoxicity enhanced by DTT treatment, whereas DTT deleted serum interference. Unfortunately, no more donor cells were available to perform flow-cytometry crossmatch, especially to detect RTX adsorption onto donor lymphocytes after a pronase treatment that removes CD20 from B-cells and so the RTX binding [7].

RTX, a CD20 monoclonal antibody, has been shown to be beneficial in some diseases such as hematopoietic malignancies, rheumatoid arthritis, autoimmune disease, and organ acute rejection. Via its Fab fragment, RTX is able to link with CD20, the receptor present on the surface of the majority of B-cells. This RTX-CD20 complex is not internalized inside the cells and the RTX Fc fragment can generate immune effect functions, leading to B-cells lysis secondary after binding a C1q fragment. RTX is also slowly eliminated from the blood circulation (half-life about 20–30 days) [8]. These pharmacokinetic properties can explain the positive CDC-XM observed in this case.

In conclusion, this case highlights the fact that recent donor RTX therapy can induce false-positive Class II CDC-XM on pretransplantation screening and reminds us that a positive crossmatch may be due to reasons related to the donor and not only to the recipient.

## Figures and Tables

**Table 1:** Prospective crossmatch performed by complement-dependent cytotoxicity for pretransplantation screening.

Sera collected months before CDC-XM	Class I results (ASHI score)		Class II results (ASHI score)	
	Without DTT	With DTT	Without DTT	With DTT
*50*	1	1	*1*	*6*
*35*	1	1	*4*	*6*
*26*	1	1	*4*	*8*
*18*	1	1	*4*	*8*
*11*	1	1	*2*	*8*
*8*	1	2	*2*	*4*
*3*	1	2	*4*	*8*
*Day of organ harvesting*	1	2	*1*	*8*

## References

[1] Guillaume N., Mazouz H., Piot V., Presne C., Westeel P.-F. (2013). Correlation between Luminex donor-specific crossmatches and levels of donor-specific antibodies in pretransplantation screening. *Tissue Antigens*.

[2] Alheim M., Paul P. K., Hauzenberger D.-M., Wikström A.-C. (2015). Improved flow cytometry based cytotoxicity and binding assay for clinical antibody HLA crossmatching. *Human Immunology*.

[3] Schlaf G., Pollok-Kopp B., Schabel E., Altermann W. (2013). Artificially positive crossmatches not leading to the refusal of kidney donations due to the usage of adequate diagnostic tools. *Case Reports in Transplantation*.

[4] Visentin J., Vigata M., Daburon S. (2014). Deciphering complement interference in anti-human leukocyte antigen antibody detection with flow beads assays. *Transplantation*.

[5] Milongo D., Vieu G., Blavy S. (2015). Interference of therapeutic antibodies used in desensitization protocols on lymphocytotoxicity crossmatch results. *Transplant Immunology*.

[6] Book B. K., Agarwal A., Milgrom A. B. (2005). New crossmatch technique eliminates interference by humanized and chimeric monoclonal antibodies. *Transplantation Proceedings*.

[7] Bearden C. M., Agarwal A., Book B. K. (2004). Pronase treatment facilitates alloantibody flow cytometric and cytotoxic crossmatching in the presence of rituximab. *Human Immunology*.

[8] Feugier P. (2015). A review of rituximab, the first anti-CD20 monoclonal antibody used in the treatment of B non-Hodgkin's lymphomas. *Future Oncology*.

